# "Transition to Clinical Years" Podcast Series: A Pilot Project to Support Rising Third-Year Students

**DOI:** 10.7759/cureus.82359

**Published:** 2025-04-16

**Authors:** Karen W Price, Harsha Bhagtani, Sofia Abraham-Hardee, Douglas Yeager, Ramu Anandakrishnan

**Affiliations:** 1 Clinical Affairs, Edward Via College of Osteopathic Medicine, Blacksburg, USA; 2 Pediatrics, Edward Via College of Osteopathic Medicine, Blacksburg, USA; 3 Clinical Affairs/Pediatrics, Edward Via College of Osteopathic Medicine, Blacksburg, USA; 4 Biomedical Sciences, Edward Via College of Osteopathic Medicine, Blacksburg, USA

**Keywords:** clinical rotation, medical education, medical student, podcast, quality improvement

## Abstract

This pilot study assessed the feasibility of podcasts as a teaching tool to support medical students for the transition to clinical training. Podcasts offer a flexible educational resource for students, allowing students to listen to content on their own time. There has been an increase in the use of podcasts for medical education and evidence that it may boost academic performance, but there are few resources available for teaching the soft skills of medicine, such as time management, adaptability, and problem solving. To address this gap, four faculty members at Edward Via College of Osteopathic Medicine (Virginia Campus) developed a podcast series with four short (10-15 minute) episodes covering non-clinical skills, including time management, clinical rotation tips, test-taking strategies, and the transition to clinical years. Students were invited via email to voluntarily complete anonymous pre- and post-intervention surveys using a Likert scale to assess their confidence and preparedness. Since Likert-scale data is ordinal and non-parametric, and as survey responses were not matched by participant, a Mann-Whitney U test was used to analyze differences between pre- and post-intervention responses. The number of participants ranged from 6 to 12 per episode. Preliminary data showed an overall increase in average scores, rising from 3.12 to 3.67 on a 1-5 scale, with statistically significant improvements in the episodes on the transition to clinical years and time management (p < 0.05). These findings suggest that podcasts may serve as a valuable supplemental tool in medical education, though further research with larger sample sizes is needed.

## Introduction

Medical education has rapidly evolved with advancements in technology, and the integration of innovative teaching modalities has become imperative to equip future physicians. Digital tools provide students with greater flexibility, faster access to information, and the ability to learn asynchronously [1]. However, the overwhelming availability of information presents a challenge, requiring physicians to develop strategies for effective lifelong learning and patient care [2]. Podcasts have emerged as a popular digital learning tool. They enable medical trainees to learn at their own pace, access expertise from physicians that may not otherwise be available to them, and facilitate a positive, low-stress learning environment [3-5]. One survey with medical residents suggested that emergency medicine residents prefer listening to podcasts (70.3%) as opposed to reading textbooks (54.3%) or reading journals (36.5%) [6]. Another survey of 356 emergency medicine residents found that 88% of emergency medicine residents reported listening to medical education podcasts at least once a month [7]. Podcasts are becoming a desirable supplemental resource for medical trainees due to increased flexibility and accessibility compared to traditional methods, such as reading a textbook. For example, students can listen to a podcast while commuting, exercising, or multitasking. Although widely used, podcasts' effectiveness in medical education varies by context; some studies show benefits in knowledge retention and performance, while others find outcomes comparable to traditional methods [3,8,9]. For example, one study demonstrated that residents’ correct answer percentage increased after listening to a topic-specific podcast (from 86% to 92% and 69% to 92%) [10]. Another study assessing knowledge retention found that medical students’ average test scores improved by 20% when tested two weeks after listening to a podcast (n=56, p<0.001) [11]. 

While numerous studies support podcasts as an effective supplement to medical education, they primarily focus on high-yield medical knowledge rather than soft skills such as time management, adaptability, and professional communication. These skills, although crucial for medical trainees, are challenging to teach through conventional methods because they often require experiential learning, mentorship, and reflection rather than direct instruction [12]. Medical students transitioning to clinical training frequently struggle with adjusting to new environments, balancing heavy workloads, understanding expectations at each rotation site, and feeling uncertain about their professional role [12,13]. These challenges are further exacerbated for students who lack access to personal mentorship. Research shows that one in five medical students has a parent who is a physician, providing them with built-in guidance and networking opportunities that their peers may not have [14]. 

Podcasts offer a new pedagogical approach that can increase access to mentorship and allow a space for medical professionals to mentor multiple students at once. One study was conducted in which four podcasts were developed for 135 preclinical medical students preparing for clinical rotations. Most students reported that the podcasts and follow-up live sessions enhanced their learning (100% and 98% of students who completed the post-curriculum survey, respectively). The study revealed that students learned strategies for engaging in productive inter-professional conversations, the use of respectful language, and the value of learning from other health professionals [15]. However, limited research has examined podcasts as a tool for teaching soft skills such as time management and clinical adaptability. 

This podcast series was developed to provide additional guidance on the transition to clinical training and address potential stressors associated with this critical period. At Edward Via College of Osteopathic Medicine (Virginia Campus), many students relocate for clinical rotations, making it more difficult to seek in-person mentorship from faculty. By offering accessible, structured advice on essential non-clinical skills, this project aims to explore the role of podcasts in bridging this gap while contributing to the broader literature on digital learning in medical education. 

## Materials and methods

This Quality Improvement Initiative was reviewed and determined to not meet the criteria for human subjects research by the Edward Via College of Osteopathic Medicine Institutional Review Board. The IRB # is 895229-1. There was no funding for this study. Informed consent was not required due to the nature of this project, but participants were informed about the study objectives and voluntary nature. 

Participants from the Edward Via College of Osteopathic Medicine (VCOM), Blacksburg, VA, were voluntarily recruited for this project through a standardized email. An initial email was sent by the Director of 3rd Year Rotations to the entirety of the class in July. A reminder email was sent approximately a month later to the class. The podcasts were piloted among the Class of 2023 students before they started their fourth year of medical school. Additional participants were recruited from the Class of 2024, 2025, and 2026 at the beginning of their third year of medical school. Each class had approximately 180 students, and there were between six and 12 participants for each of the four episodes. 

Podcast topics were selected based on faculty-identified gaps in the curriculum, informed by discussions with third-year medical students who scored below 75% on an end-of-rotation exam and reported challenges during transitions. The topics of the podcasts included 1) Transition to Clinical Years Overview, 2) Clinical Rotation Tips, 3) Test Taking Tips, and 4) Time Management. Each podcast was about 10-15 minutes long to encourage participation and maximize the learner’s experience. One survey of Canadian anesthesiology residents reported that listeners preferred a 5- to 15-minute length for teaching modalities such as case discussions, debates, and journal article summaries [9]. The podcasts and surveys were posted on the students’ course website so they could listen and complete the surveys at their discretion. 

Students who opted to listen to the podcasts were asked to complete anonymous pre- and post-surveys with the goal of giving feedback to improve the podcasts for future students. The pre- and post-surveys consisted of five questions each and were designed to assess students’ confidence in their responsibilities in their next step of training. They included statements about understanding what material to expect on an end-of-rotation exam, knowing what educational resources to use, and effectively managing time. While these surveys were not externally validated, they were developed based on faculty expertise and student feedback. There are no standardized or widely accepted surveys for podcasts. This pilot project will help validate this survey for future studies. Faculty members had discussions with students who scored less than 75% on an end-of-rotation exam, which helped to gauge their challenges in their transitions to clinical training. Images of the pre- and post-intervention surveys are provided in the Appendix (Figures 5-12). A Likert scale was used for participants to answer each statement. This scale was selected to identify how much a person agreed or disagreed with each statement. A 5-point scale was used ranging from strongly disagree to strongly agree, with each answer correlating to a number 1-5 to allow for descriptive statistical analysis. The responses for each pre- and post-surveys were not linked in this preliminary study. The responses were not linked because there was a separate pre- and post- intervention survey for each episode (a total of eight surveys). To link the surveys, identifying information, such as email or name, would need to be obtained. However, to preserve confidentiality, identifying information was not documented. 

The data from the survey was analyzed using descriptive statistics, mainly mean, to quantify survey responses and compare the pre- and post-intervention responses to each question. The Mann-Whitney U test was used to compare the pre- and post-intervention responses averaged across all questions for each participant. The test was chosen due to the small sample sizes and the ordinal nature of the Likert scale data. 

## Results

**Figure 1:**
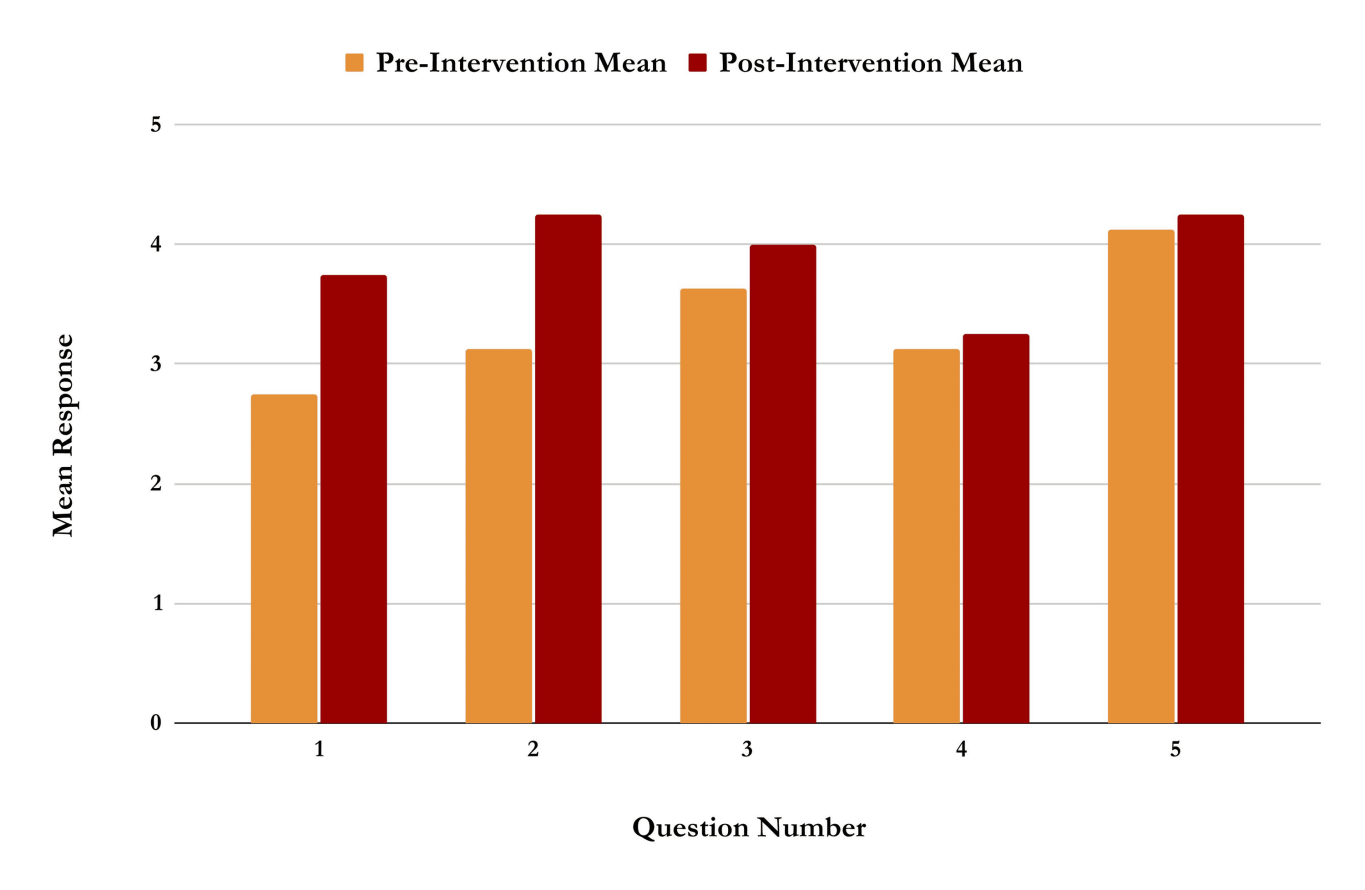
Graph of responses to each of the five questions on the pre- and post-intervention survey for the podcast episode titled “Transition to Clinical Years Overview”. N=12. There was an increase in mean survey response across all survey questions.

Data were collected via a Google Survey and Google Spreadsheet. For each podcast episode, the mean pre- and post-intervention responses across all five survey questions were analyzed using the Mann-Whitney U test to determine statistical significance. For Podcast Episode 1 (Figure 1), titled “Transition to Clinical Years Overview” (n=12), mean responses increased across all questions, with the largest increase observed in Question 1 (from 2.75 to 3.75). This question assessed participants' feelings of readiness and knowledge of what material to expect on an end-of-rotation exam. An increase in one point is the equivalent of going from neutral to agree, or from slightly agree to strongly agree. The average participant score for this episode increased from 3.35 to 3.90 on a scale of 1-5, suggesting a meaningful gain in confidence after listening to the podcast. The Mann-Whitney U test yielded a U-value of 4, with a z-score of -1.67115 and a p-value of 0.04746, indicating a statistically significant difference at p < 0.05. 

**Figure 2:**
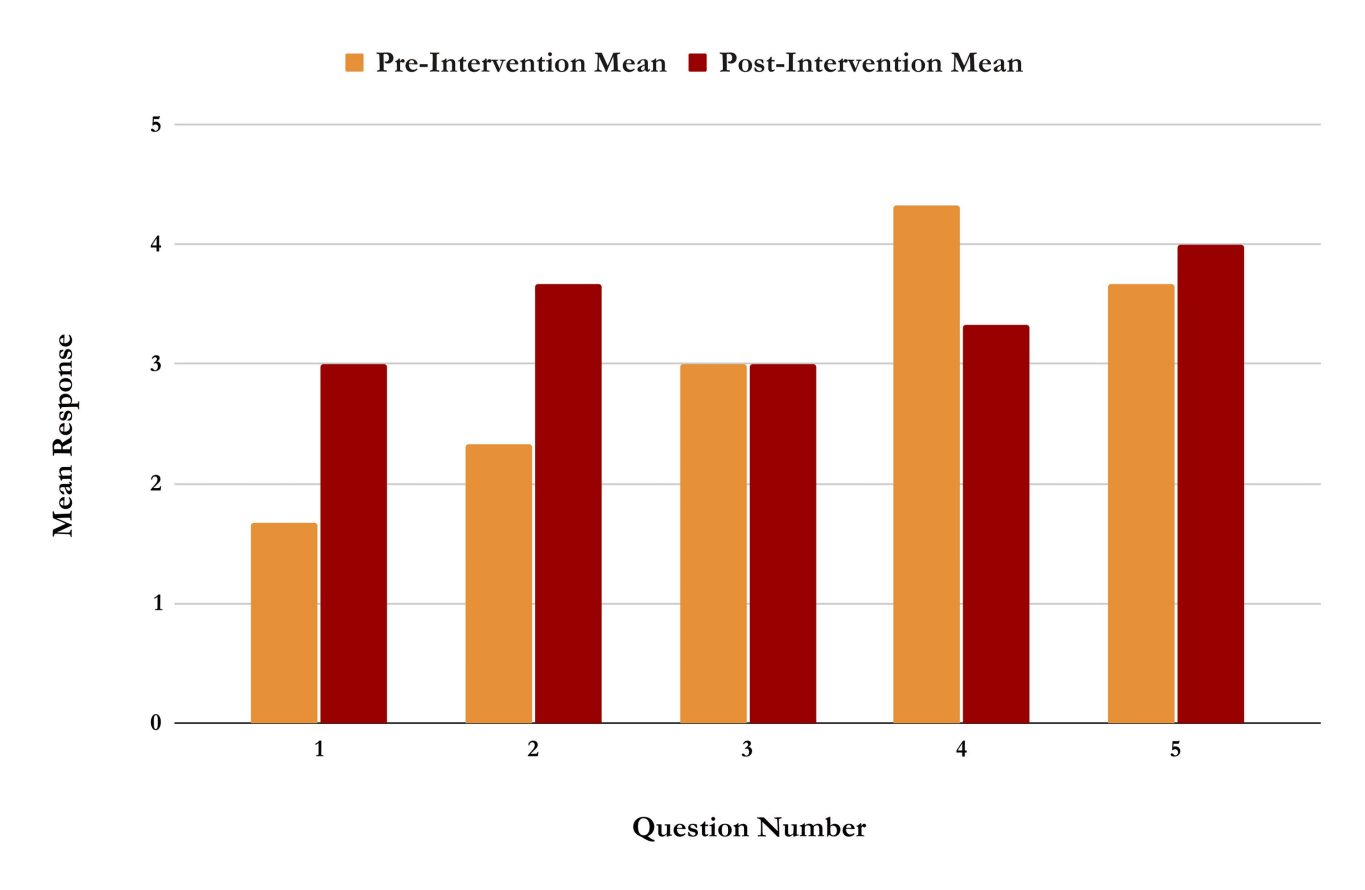
Graph of responses to each of the five questions on the pre- and post-intervention survey for the podcast episode titled “Clinical Rotation Tips”. N=6. There was an increase in mean survey response for questions 1, 2, and 5. There was no change in mean for question 3, and a decrease in the mean for question 4.

For Podcast Episode 2 (Figure 2), titled “Clinical Rotation Tips” (n=6), mean responses increased for most questions, except for question 3, which did not change, and question 4, which decreased. The average participant score for this episode increased from 3.00 to 3.40 on a scale of 1-5, suggesting a meaningful gain in confidence after listening to the podcast. However, the Mann-Whitney U test yielded a U-value of 9.5, with a z-score of -0.52223 and a p-value of 0.30153, indicating no statistically significant difference at p < 0.05. 

**Figure 3:**
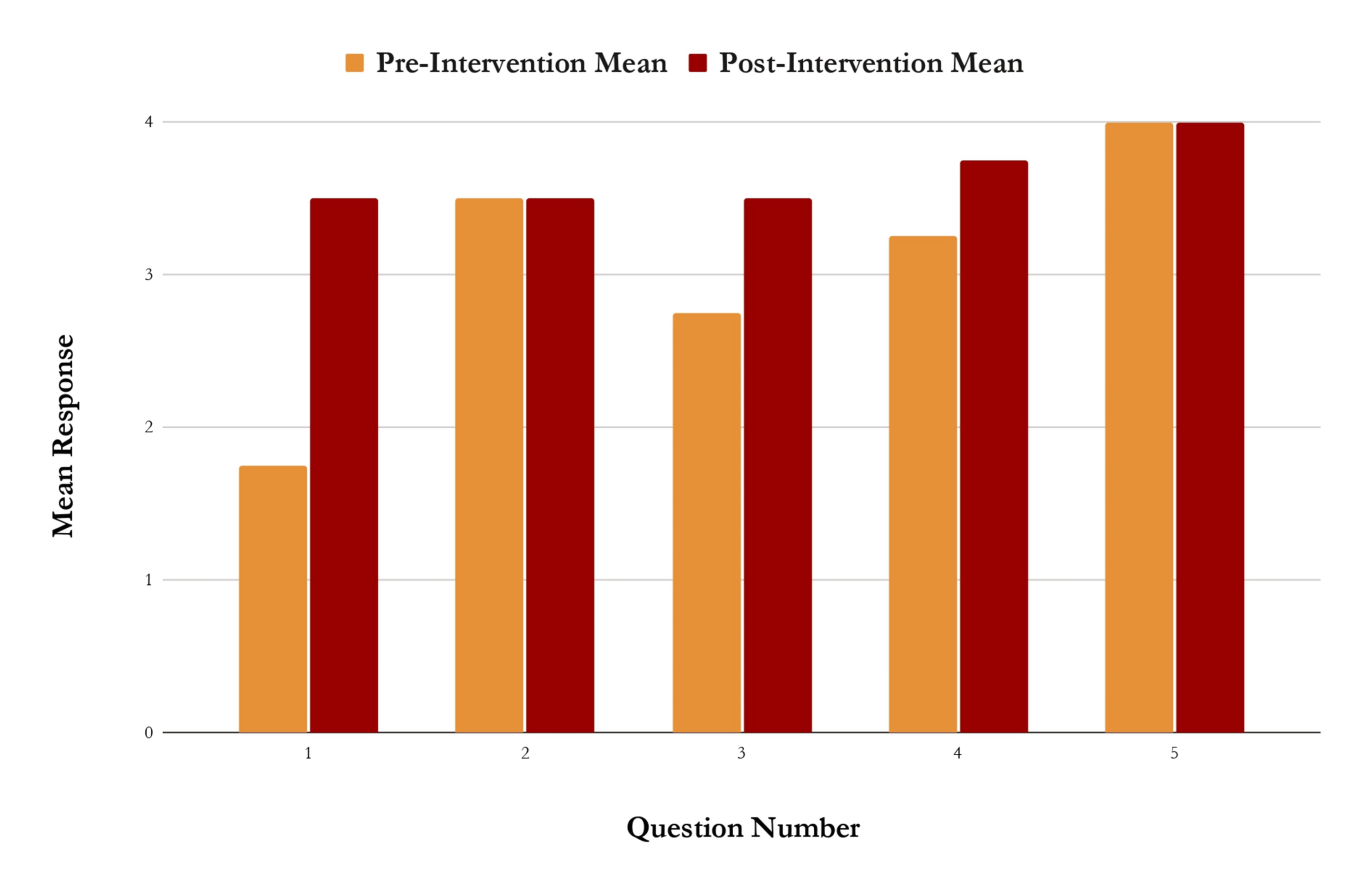
Graph of responses to each of the five questions on the pre- and post-intervention survey for the podcast episode titled “Test Taking Tips”. N=8. There was an increase in mean survey response for questions 1, 3, and 4. There was no change in the mean survey response for questions 2 and 5.

For Podcast Episode 3 (Figure 3), titled “Test Taking Tips” (n=8), mean responses increased for most questions, with Question 1 showing the largest improvement (from 1.75 to 3.50). Notably, this question covered the same topic as Question 1 from Episode 1-readiness and expectations for end-of-rotation exams. The average participant score for this episode increased from 3.05 to 3.65 on a scale of 1-5, suggesting a meaningful gain in confidence after listening to the podcast. The Mann-Whitney U test yielded a U-value of 6, with a z-score of -1.25336 and a p-value of 0.10565, indicating no statistically significant difference at p < 0.05. 

**Figure 4:**
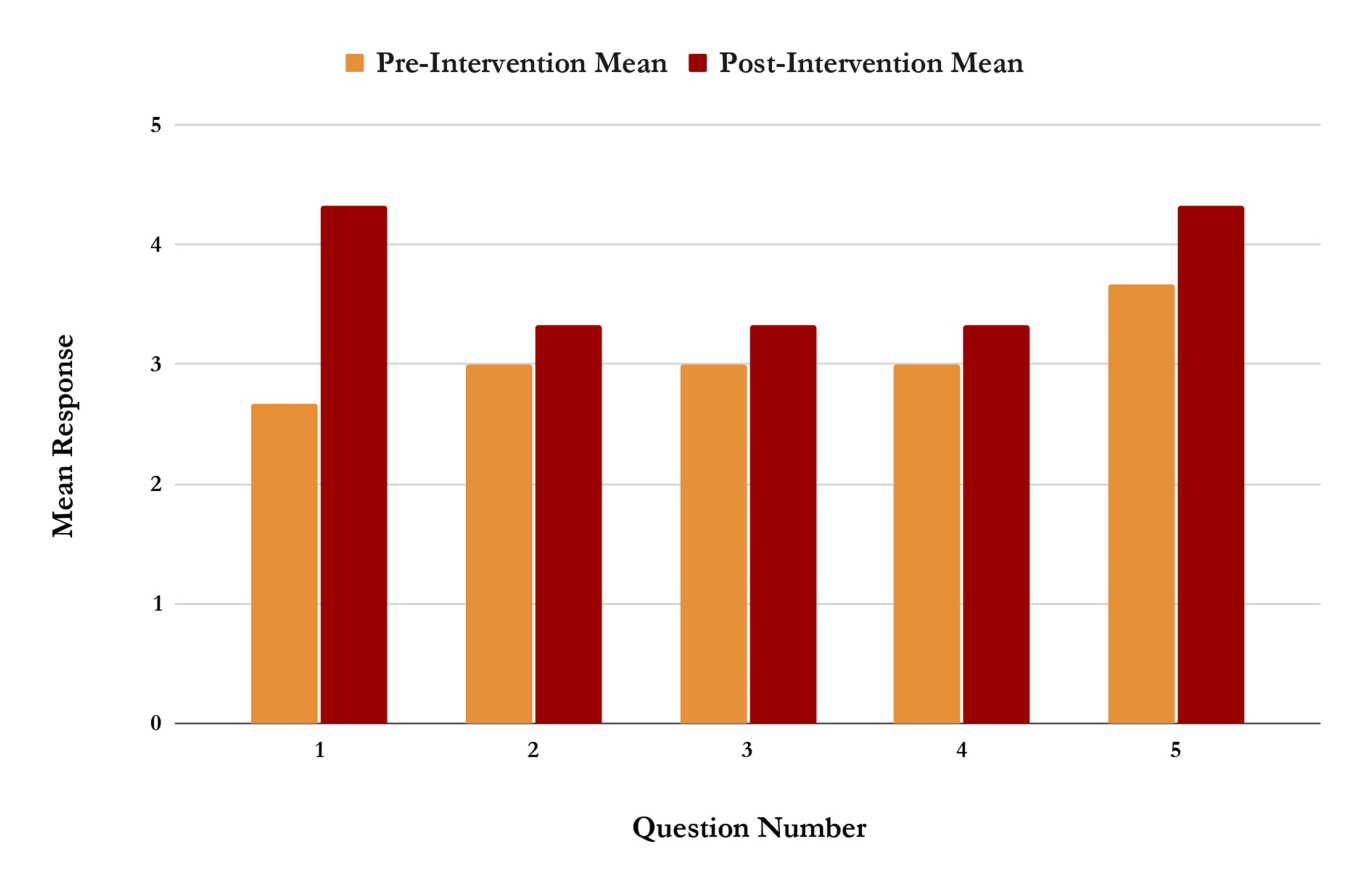
Graph of responses to each of the five questions on the pre- and post-intervention survey for the podcast episode titled “Time Management”. N=6. There was an increase in mean survey response across all survey questions.

**Figure 5:**
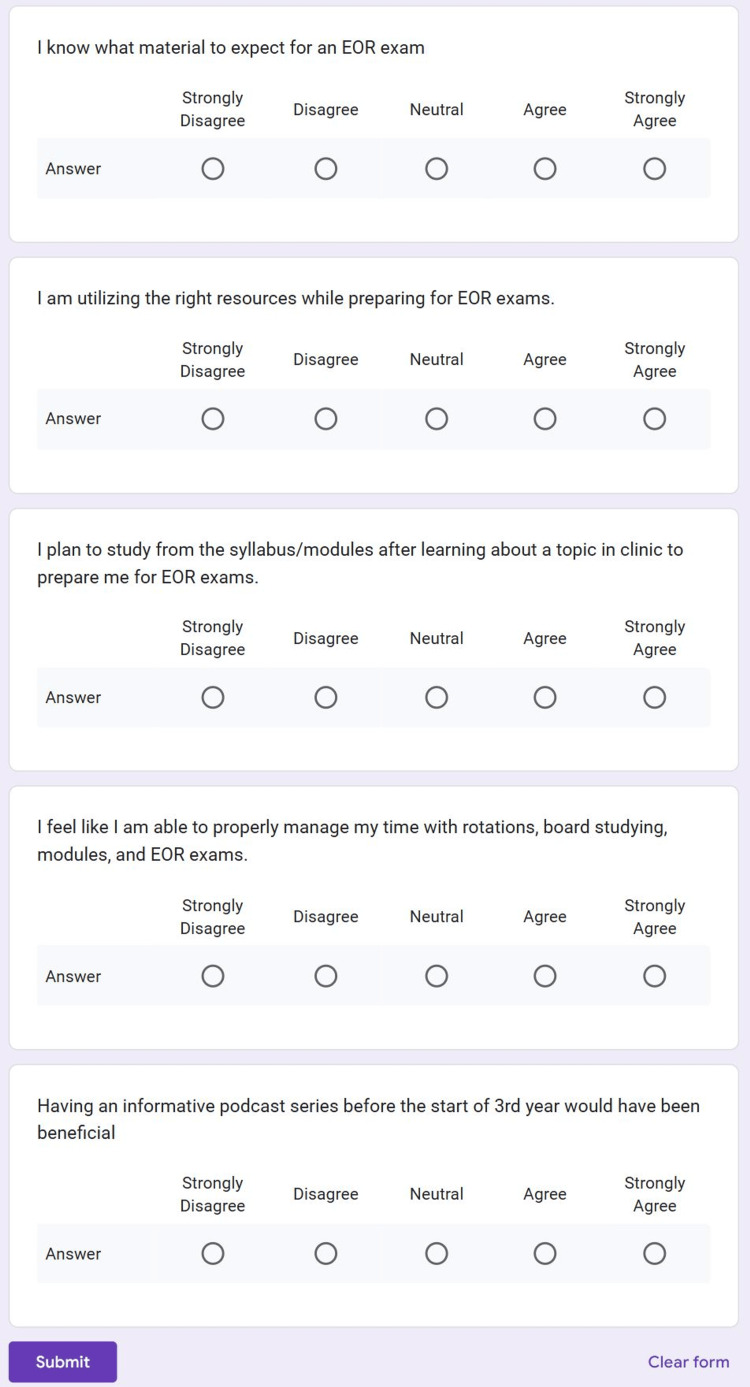
Pre-Intervention Survey for "Transition To Clinical Years Overview" Podcast Episode EOR: End of Rotation

**Figure 6:**
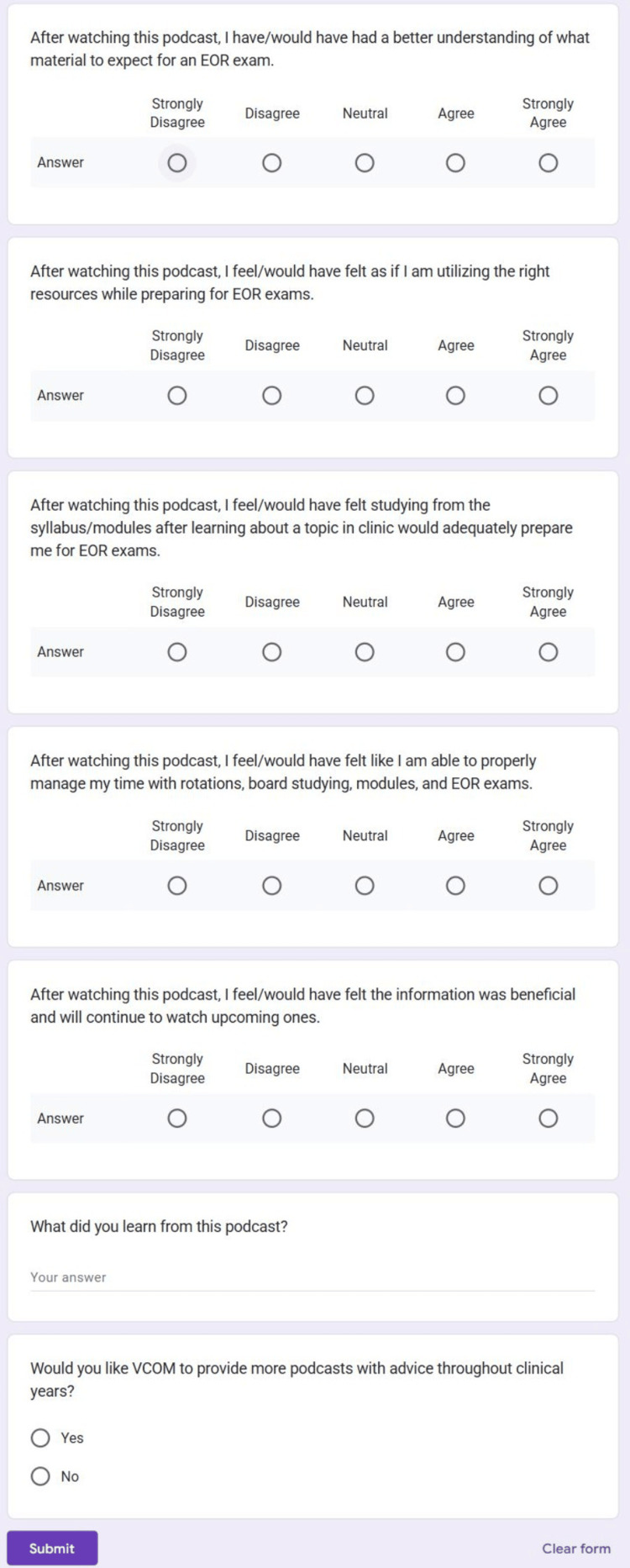
Post-Intervention Survey for "Transition To Clinical Years Overview" Podcast Episode EOR: End of Rotation

**Figure 7:**
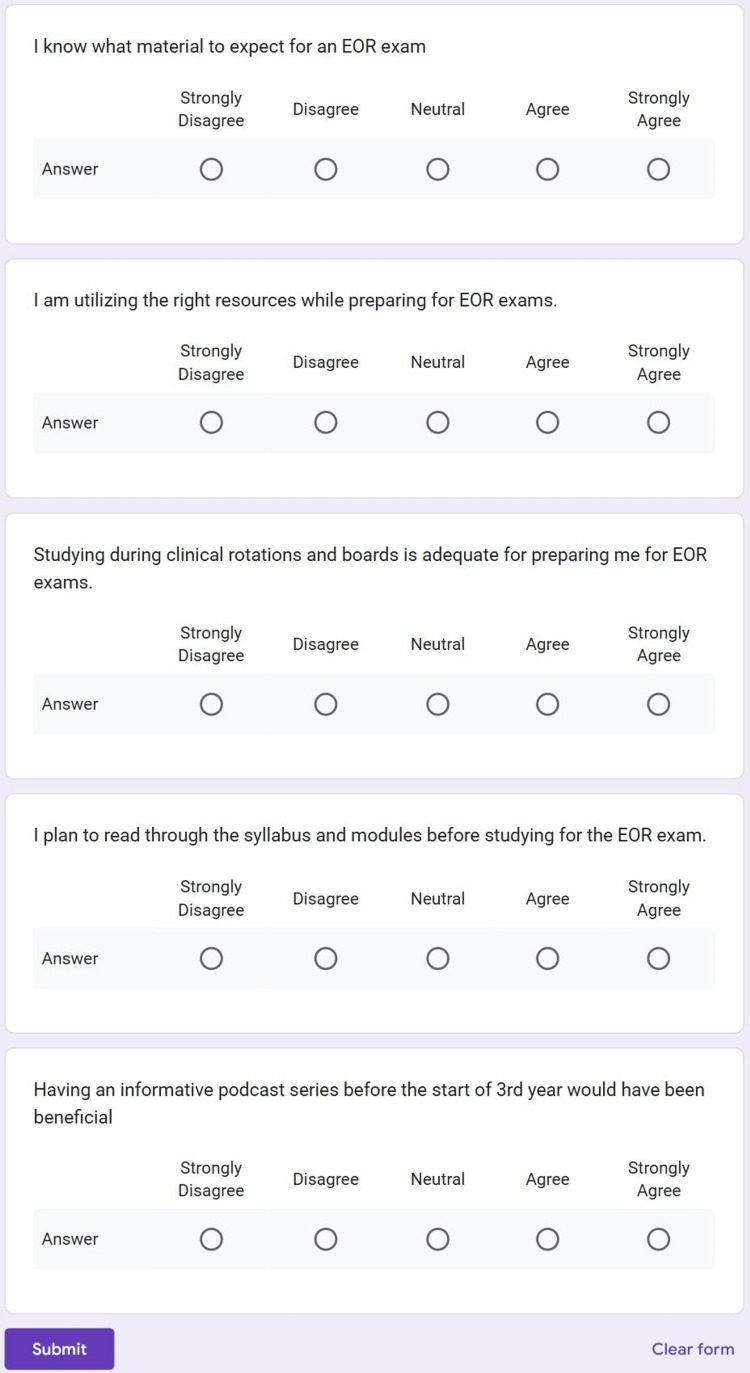
Pre-Intervention Survey for "Clinical Rotation Tips" Podcast Episode EOR: End of Rotation

**Figure 8:**
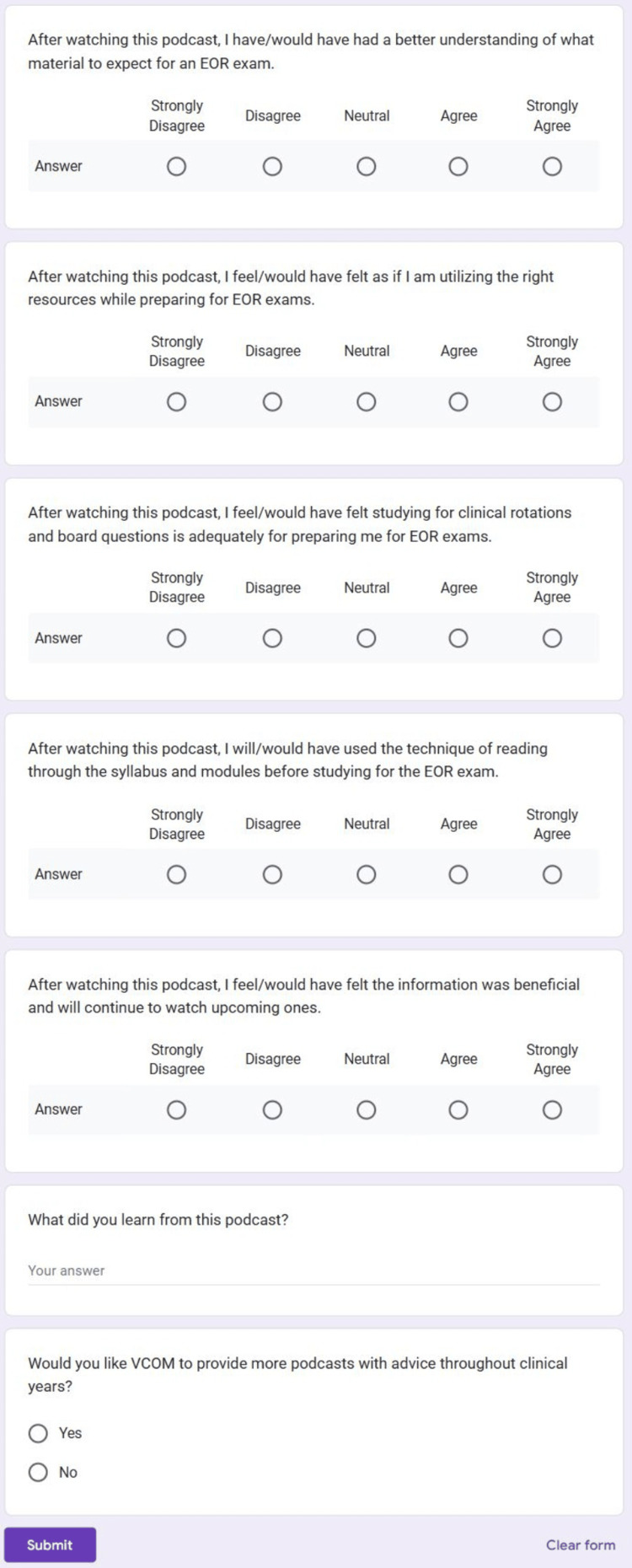
Post-Intervention Survey for "Clinical Rotation Tips" Podcast Episode EOR: End of Rotation

**Figure 9:**
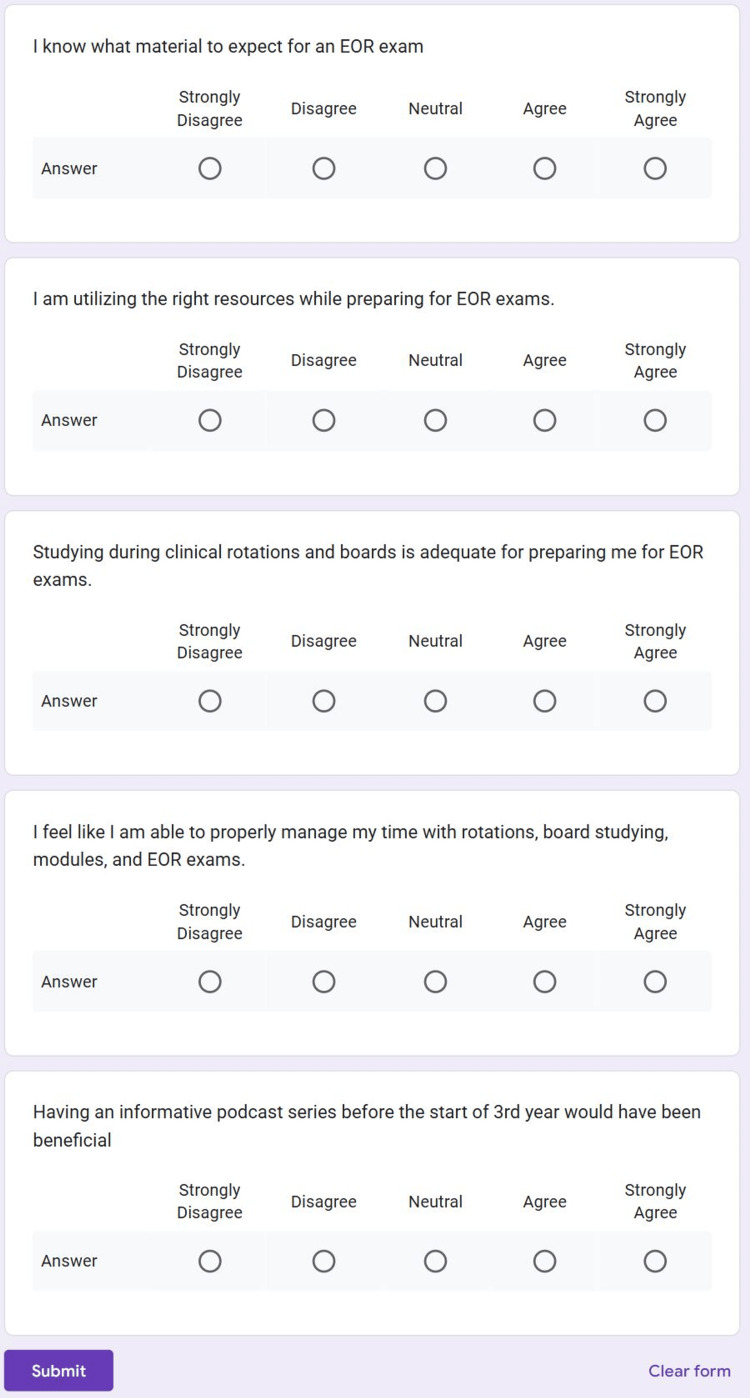
Pre-Intervention Survey for "Test Taking Tips" Podcast Episode EOR: End of Rotation

**Figure 10:**
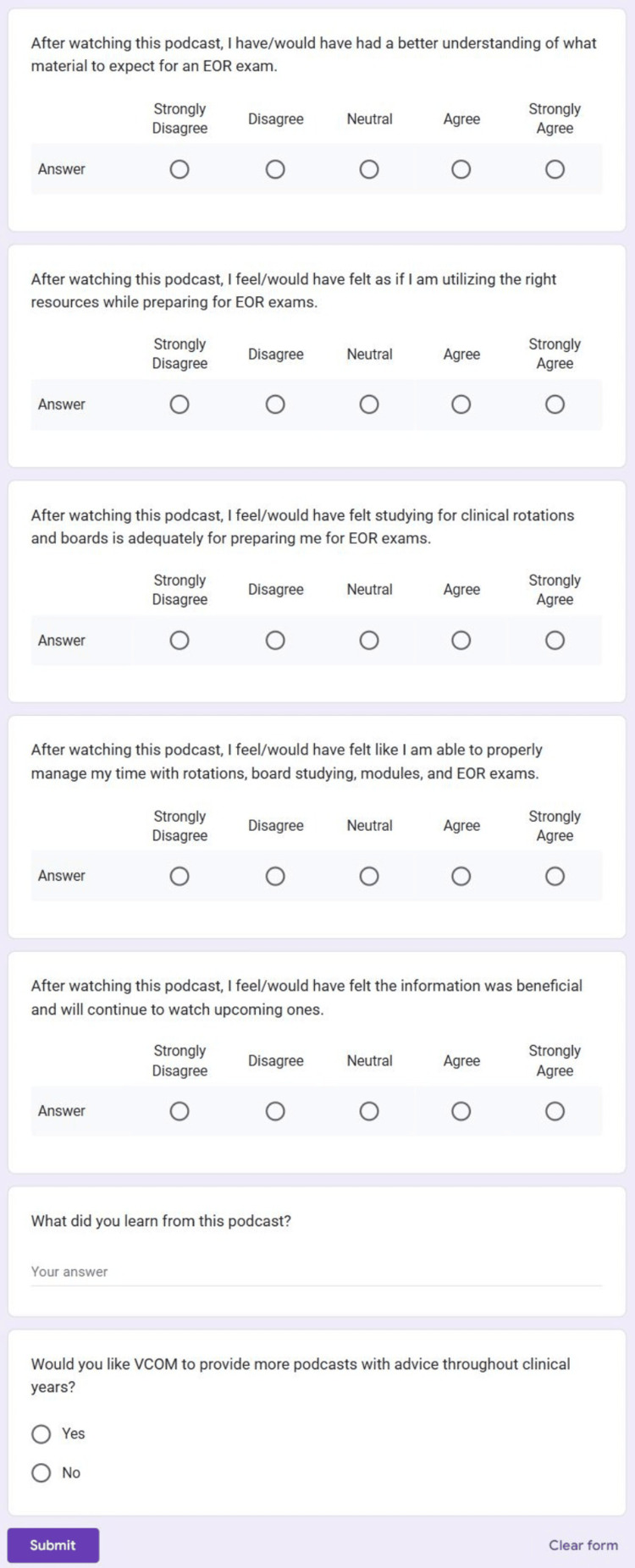
Post-Intervention Survey for "Test Taking Tips" Podcast Episode EOR: End of Rotation

**Figure 11:**
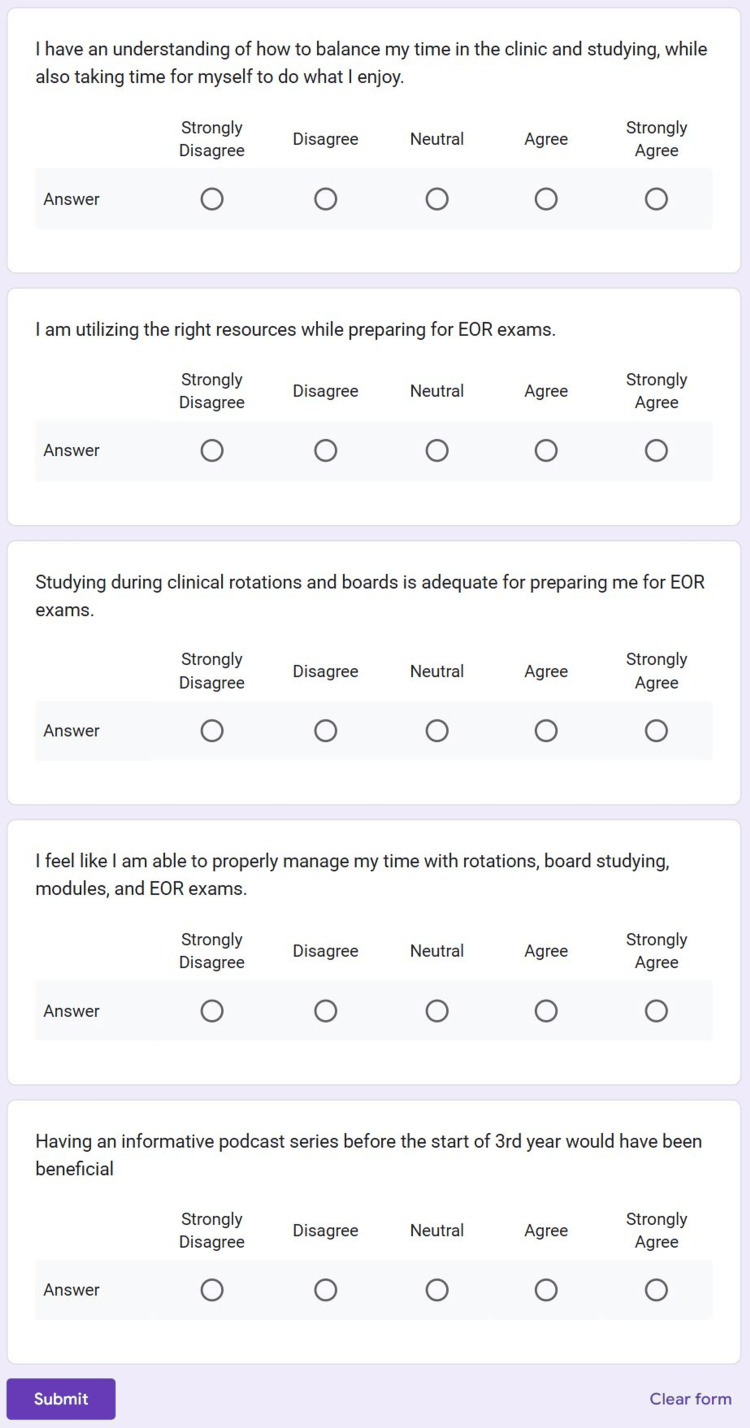
Pre-Intervention Survey for "Time Management" Podcast Episode EOR: End of Rotation

**Figure 12:**
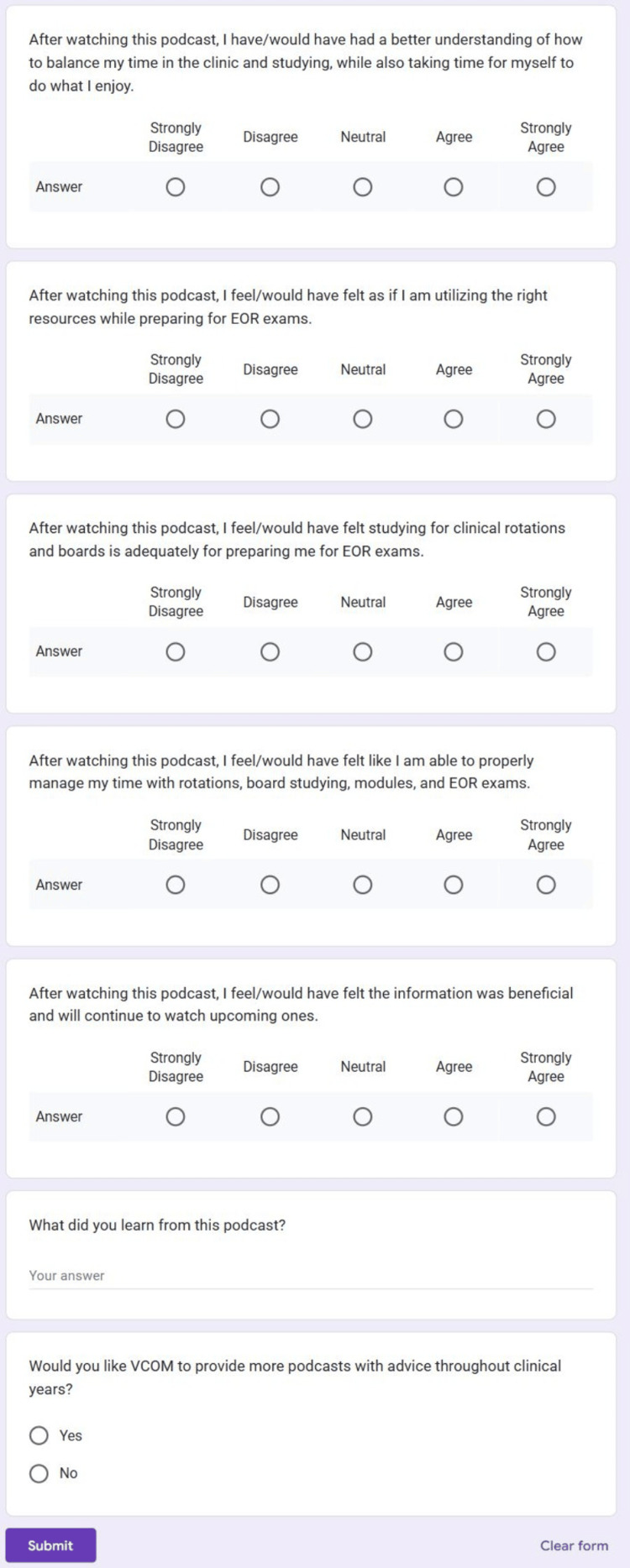
Post-Intervention Survey for "Time Management" Podcast Episode EOR: End of Rotation

For Podcast Episode 4 (Figure 4), titled “Time Management” (n=6), mean responses increased across all questions, with Question 1 showing the largest improvement (from 2.67 to 4.33). The average participant score for this episode increased from 3.07 to 3.73 on a scale of 1-5, suggesting a meaningful gain in confidence after listening to the podcast. The Mann-Whitney U test yielded a U-value of 3, with a z-score of -1.88004 and a p-value of 0.03005, indicating a statistically significant difference at p < 0.05. An additional yes or no question was listed at the end of each post-intervention survey, which asked if the participant would like the institution to provide more podcasts with advice throughout clinical years. Across all four surveys, 100% (n=14) of participants responded “yes.” 

## Discussion

Preliminary data showed an overall increase in average scores, rising from 3.12 to 3.67 on a 1-5 scale, with statistically significant improvements in the episodes on the transition to clinical years and time management (p < 0.05). However, the episodes on clinical rotation tips and test taking tips showed trends of improvement that did not reach statistical significance. One possible explanation for this discrepancy is the variation in sample sizes, as Podcast Episodes 1 and 3 had larger participation (n=12 and n=8, respectively) compared to Episodes 2 and 4 (both n=6). With such small sample sizes, the power to detect statistically significant differences is inherently limited. Additionally, topic relevance may have played a role in engagement levels. The third episode focused on exam readiness, a topic of high importance to medical trainees, which could have influenced both participation rates and perceived impact. Another factor that may have contributed to the variability in participation was the placement of survey links on the course page. The survey for Podcast Episode 1 was listed first, which may have led some participants to complete only the first set of surveys, mistakenly assuming that additional surveys were not required. Due to the confidentiality of responses, we were unable to track whether individuals who completed the first survey also participated in subsequent surveys. This may have further impacted the statistical power of the later episodes, limiting the ability to detect significant changes in responses. To address these challenges in future iterations, practical strategies could include adding QR codes at the beginning of each episode to streamline survey access, consolidating the surveys into a single post-intervention form to reduce survey fatigue, or sending reminders at standardized intervals to increase response rates.

The observed increases in mean scores suggest that participants found the podcast series beneficial, particularly in improving their understanding of clinical expectations and time management strategies. While statistical significance provides one measure of impact, educational significance must also be considered. Even in cases where results were not statistically significant, the upward trends indicate that learners may have gained useful insights. Prior research on podcasts in medical education has demonstrated mixed results, with some studies showing improvements in knowledge retention and confidence, while others indicate comparable outcomes to traditional learning methods. For example, studies have found that podcasts can improve test performance and knowledge retention, yet their effectiveness varies depending on content delivery, learner engagement, and integration with other educational strategies [3,8,9]. 

The post-intervention survey also welcomed student feedback regarding the strengths and areas for improvement in the podcast series. Positive takeaways included learning how to apply clinical experiences to exam preparation and recognizing the value of learning directly from patient interactions. However, participants also identified areas for improvement, particularly regarding end-of-rotation exam expectations. One student expressed continued stress due to the variability of rotation sites despite standardized exams, suggesting that future podcast episodes could provide more detailed guidance on navigating these inconsistencies. Addressing these concerns by incorporating faculty insights or student testimonials from different rotation sites may enhance the utility of the series. A formal needs assessment could also help identify the most relevant topics for students, ensuring that podcast content aligns with learner priorities.

Despite the overall positive response, this study did not assess objective performance metrics, such as improvements in test scores or clinical evaluations. Instead, findings primarily reflect subjective measures of confidence and perceived preparedness. Previous studies have demonstrated that while podcasts can supplement medical education, their standalone effectiveness is variable [10,11]. The fact that participants reported increased confidence, but not necessarily improved performance aligns with findings that suggest podcasts are most effective when used alongside active learning methods rather than as a sole instructional tool.

Limitations

The greatest limitation of this study is the low participation rate. We speculate that the small number of responses was due to a lack of participation and failure to complete all of the surveys. The number of respondents differed across episodes, with the highest participation in the first episode (n=12) and the lowest in the second and fourth episodes (n=6). This variability may have influenced the statistical power of our findings, limiting the ability to detect significant differences in certain episodes. Additionally, the placement of survey links on the course page may have contributed to an uneven distribution of responses, as the first episode’s survey was listed first, potentially leading some participants to complete only that set of surveys. Due to confidentiality, we could not track individual participation across multiple episodes, making it difficult to assess whether engagement decreased over time. 

Another limitation was the lack of survey validation and inability to link pre- and post-survey responses at the individual level. If the surveys were validated, it may have allowed more comprehensive feedback on the impact of the podcast series. There are no standardized surveys for podcasts, so this pilot study will help to validate this survey for future studies. Also, since survey data were anonymous, analyses were conducted using overall pre- and post-survey data rather than paired responses. This prevented more detailed statistical comparisons that could have provided a clearer picture of individual improvements. Future studies could address this by assigning unique participant identifiers to track changes while maintaining anonymity. Alternatively, the surveys could include a QR code to facilitate survey completion or consolidate to have only a post-intervention survey, which would reduce survey fatigue. Additionally, this study relied on self-reported measures of confidence and perceived preparedness rather than objective performance outcomes, such as test scores or clinical evaluations. While subjective confidence is an important factor in student success, future research should assess whether increased confidence translates into improved academic or clinical performance. Finally, voluntary participation introduces the possibility of self-selection bias, as students who choose to engage with the podcast and complete surveys may have been more receptive to this learning format. This limits the generalizability of the findings, as students with different learning preferences or levels of prior knowledge may have responded differently. 

## Conclusions

This study highlights a gap in medical education and demonstrates the potential of podcasts as a supplemental teaching tool for addressing the transition to clinical training. While results showed an overall increase in average scores from 3.12 to 3.67 on a 1-5 scale between the pre- and post-intervention surveys, statistical significance was only observed in two podcast episodes, likely due to the small sample size and variability in participant engagement. Nevertheless, the upward trends and positive student feedback suggest that learners found value in the podcast series, particularly in areas such as time management and understanding clinical expectations.

As medical education continues to evolve with digital learning tools, podcasts represent a promising avenue for bridging knowledge gaps and providing accessible mentorship during critical transitions in training. Integrating podcasts into formal curricula or pairing them with interactive discussions may further optimize their educational value, making them a sustainable and impactful resource for medical trainees.
